# Local Guidance on the Management of Nephropathic Cystinosis in the Gulf Cooperation Council (GCC) Region

**DOI:** 10.3390/children12080992

**Published:** 2025-07-28

**Authors:** Hassan Aleid, Turki AlShareef, Ahmad Kaddourah, Maryam Zeinelabdin, Mohamad M. Alkadi, Badria Al Ghaithi, Yasser Abdelkawy, Eihab Al Khasawneh, Elena Levtchenko, Khalid Alhasan

**Affiliations:** 1 Department of Kidney and Pancreas Transplant, King Faisal Specialist Hospital and Research Centre, Riyadh P.O. Box 3354, Saudi Arabia; heid@kfshrc.edu.sa; 2 Department of Pediatrics, King Faisal Specialist Hospital and Research Centre, Riyadh P.O. Box 3354, Saudi Arabia; tshareef@kfshrc.edu.sa; 3 Sidra Medical and Research Centre, Doha P.O. Box 26999, Qatar; akaddourah@sidra.org; 4 Weill Cornell Medicine-Qatar, Doha P.O. Box 24811, Qatar; malkadi@hamad.qa; 5 College of Medicine, Qatar University, Doha P.O. Box 2713, Qatar; 6 Division of Nephrology, Department of Pediatrics, East Jeddah General Hospital, Jeddah 8742, Saudi Arabia; mzeinelabdin@moh.gov.sa; 7 Division of Nephrology, Department of Medicine, Hamad Medical Corporation, Doha P.O. Box 3050, Qatar; 8 Department of Child Health, Royal Hospital, Muscat P.O. Box 1331, Oman; badria.alghaithi@gmail.com; 9 Ministry of Health, P.O. Box 43787, Hawalli 32052, Kuwait; kawyaser@yahoo.com; 10 Division of Pediatric Nephrology, Sheikh Khalifa Medical City, Abu Dhabi 51900, United Arab Emirates; ekhasawneh@sech.sa; 11 Department of Pediatric Nephrology, Emma Children’s Hospital, Amsterdam University Medical Centre, 1105 AZ Amsterdam, The Netherlands; e.n.levtchenko@amsterdamumc.nl; 12 Pediatric Department, College of Medicine, King Saud University, P.O. Box 800, Riyadh 11421, Saudi Arabia

**Keywords:** nephropathic cystinosis, cystine, cysteamine, clinical practice guidance, GCC countries

## Abstract

Cystinosis is a rare systemic disease characterized by the accumulation of cystine in tissues, leading to multi-organ damage. Infantile nephropathic cystinosis is the dominant and severe form of cystinosis with critical renal manifestations that require kidney transplantation at an early age if left untreated. Cysteamine, the lifelong cystine-depleting therapy, is the mainstay treatment of nephropathic cystinosis. Cysteamine prevents cystine crystal formation and delays disease progression. While the initially introduced cysteamine consists of an immediate-release (IR) formulation, a delayed-release (DR) formulation has been developed with a simplified dosing regimen (Q12H instead of Q6H) and an improved quality of life while maintaining comparable efficacy. Due to the rare incidence of the disease and lack of international guidelines, diagnosis and treatment initiation are oftentimes delayed, leading to a poor prognosis. Pediatric and adult nephrologists from Kuwait, Saudi Arabia, the United Arab Emirates (UAE), and Qatar, in addition to one international expert from Amsterdam, convened to share their clinical experience, reflecting on the challenges encountered and therapeutic approaches followed in the management of nephropathic cystinosis in the Gulf Cooperation Council (GCC) region. Experts completed a multiple-choice questionnaire and engaged in structured discussions, where they shed light on gaps and limitations with regard to diagnostic tests and criteria to ensure early diagnosis and timely treatment initiation. Based on available literature, experts suggested an algorithm to help guide nephropathic cystinosis management in the GCC. It is highly recommended for patients who do not tolerate IR-cysteamine and do not adhere to IR-cysteamine treatment to switch to DR-cysteamine. Given the systemic nature of the disease, a multi-disciplinary approach is required for optimal disease management.

## 1. Context

The incidence of cystinosis worldwide is estimated at one in 100,000–200,000 live births [1]. Cystinosis is an autosomal recessive lysosomal storage disorder caused by mutations in the *CTNS* gene coding for the lysosomal cystine transporter, cystinosin [1,2,3]. The defect in cystine efflux from the lysosome leads to its accumulation in the cells, increasing the cellular cystine levels to up to 800 times the normal level [1,2,3]. Cystinosis is a systemic disease affecting several organs, starting with kidneys, and multiple non-renal complications can occur over time [3,4]. Based on the age at disease manifestation and the degree of kidney disease severity, three clinical forms of cystinosis can be distinguished: infantile nephropathic cystinosis, the most common form of the disease, which accounts for about 95% of patients diagnosed with cystinosis, followed by late-onset nephropathic cystinosis, considered to be rare, and non-nephropathic ocular cystinosis, with the lowest incidence [5]. Infantile nephropathic cystinosis is the most severe form of cystinosis, which leads to kidney failure in the first decade of life if left untreated [1,3,5]. Infantile cystinosis is the most common underlying cause of inherited renal Fanconi syndrome (a generalized defect in proximal tubular reabsorption). Late-onset (intermediate or juvenile) nephropathic cystinosis has similar manifestations but generally occurs at adolescence and is associated with glomerular impairment but not necessarily Fanconi syndrome [1,3,5]. Due to its various manifestations, diagnosis is complicated and may rely on kidney failure. Finally, non-nephropathic ocular (adult or benign) cystinosis is only characterized by ocular manifestations due to corneal crystals and is associated with discomfort and high sensitivity to light (photophobia), with no kidney or other organ involvement [1,5].

Clinical symptoms usually develop as early as 6 months of age, with a failure to thrive and rickets being common manifestations of renal Fanconi syndrome. Corneal crystals, the hallmark of cystinosis, manifest at 18 months of age [6]. While chronic kidney failure is manifested between the ages of 8 to 12 years, diabetes, underactive testicles, muscle wasting, lung dysfunction and central nervous system damage present between the ages of 18 and 40 years [1].

All individuals with classical nephropathic cystinosis suffer from cystinosis metabolic bone disease (CMBD), related to the renal Fanconi syndrome in infancy and progressive chronic kidney disease (CKD) later in life. Manifestations of CMBD include hypophosphatemic rickets in infancy and renal osteodystrophy associated with CKD, resulting in bone deformities, osteomalacia, osteoporosis, fractures, and short stature [7].

Given the particular situation in the Arab world, where consanguinity is predominant [8], genetic diseases tend to manifest frequently, calling for timely and streamlined management across the region, where similar patient profiles are encountered.

## 2. Methods

Pediatric and adult nephrologists from Kuwait (1 expert), Saudi Arabia (4 experts), Qatar (2 experts), and the United Arab Emirates (UAE) (1 expert), in addition to one international expert from Amsterdam, convened to share their clinical experience in managing nephropathic cystinosis in the GCC region. They completed a 34-question multiple-choice questionnaire hosted on Google Forms and participated in live structured discussions that highlighted challenges, diagnostic gaps, and therapeutic approaches. Discussions focused on three main concepts, and insights from the panelists were summarized in the sections below. Experts responses were presented as percentages.

## 3. Nephropathic Cystinosis in the Gulf Cooperation Council

According to panelists from different countries in the Gulf Cooperation Council (GCC), an entity that is distinct from the GCC Health Council, to date, around 90 patients have been diagnosed with nephropathic cystinosis in the GCC, of whom 50 patients are in King Faysal Hospital, the main nephropathic cystinosis referral center in Saudi Arabia. This is consistent with the high consanguineous marriage rate in the Arab world, estimated at half of all marriages versus 20% in the Western world [8]. Specifically, up to 39% of all marriages are consanguineous in the UAE [9] and 62.6% in Qatar [10].

Aligned with the literature, panelists predominantly encounter the infantile nephropathic cystinosis form with Fanconi syndrome. Extra-renal manifestations are major concerns in nephropathic cystinosis and should be properly managed by specialists, according to affected organs. Around 70% reported encountering growth failure and 29% glycosuria among their patients as nephropathic cystinosis manifestations. CKD was reported as the predominant renal condition after Fanconi syndrome, according to 43% of the experts, followed by kidney transplantation (29%), while corneal crystals and hypothyroidism were extra-renal manifestations reported by 86% and 71% of the experts, respectively. Only a few experts reported encountering central nervous system manifestations and metabolic diseases, including diabetes and dyslipidemia.

## 4. Clinical Management of Nephropathic Cystinosis

### 4.1. Diagnostic Tests

Cystinosis is diagnosed based on three different modalities: by assessing white blood cell (WBC) cystine levels, corneal crystals, and genetic analysis [1,11].

Only 29% of the convened experts from the GCC perform the WBC cystine level test, due to its unavailability in most countries and centers, while 57% perform slit lamp exams for corneal cystine crystal identification, and 71% of experts check for the *CTNS* mutations. Around 40% of the experts consider the three approaches to confirm a nephropathic cystinosis diagnosis.

#### 4.1.1. WBC Cystine Level

WBC cystine assay is the current gold standard for diagnosis and treatment monitoring. Cystine level is measured either in mixed WBC or isolated granulocytes (polymorphonuclear cells) [5,12]. Given that granulocytes accumulate the most cystine in blood, measurement in this fraction provides more reliable and accurate results than mixed WBC [13]. A healthy individual presents a cystine level ≤0.5 nmol hemicystine/mg protein, while an untreated affected individual shows a cystine level >1 nmol hemicystine/mg protein. The level of cystine in WBC is considered as a biomarker for systemic disease, but it also indicates the efficacy of cystine-depleting therapy (CDT), discussed below. Treated patients with controlled cystinosis are expected to have a WBC cystine level ≤ 1 nmol hemicystine/mg protein [14], which is set as a target level in cystinosis treatment. Whether systemic cystine level reflects tissue cystine levels is still to be elucidated. Monitoring cystine levels helps CDT dose titration to establish a stable effective dose after treatment. Despite the high specificity of the assay, it is associated with many limitations. The assay is available only in a small number of laboratories, and a minimum of a 3 mL blood sample (heparinized to prevent clotting) is required to be tested within a 24 h interval after sampling due to the instability of cystine in blood cells [2,15].

WBC cystine level assessment remains limited in the GCC. Only 29% of the GCC panelists perform the WBC test due to its unavailability as agreed on by 57% of the experts, but also due to its related limitations: storage condition and time constraint between storage and test performance (within 24–72 h), as mentioned by 29% of the experts, and the required volume of blood (14% of experts).

#### 4.1.2. Slit-Lamp Examination

Corneal crystals are present early in the disease course, by 1–2 years of age. In developing countries, slit-lamp examination is considered the most adapted diagnostic method for cystinosis due to the ease and availability of the test, in addition to its lower cost compared to other methods. However, grading and identifying crystals needs a considerably experienced ophthalmologist [13].

Corneal crystals are a hallmark of the disease. However, according to GCC experts, a sole ocular manifestation with no proteinuria or any indicator of kidney damage should not be considered sufficient for further investigations on nephropathic cystinosis.

In the GCC, panelists rely on glycosuria and corneal crystals as major factors for nephropathic cystinosis diagnosis. A considerable gap between disease onset and diagnosis was reported in the region. While most patients are diagnosed fortuitously when being examined and treated for other conditions, there is concern that others are misdiagnosed with cystinosis instead of cystinuria based on elevated urine cystine level or remain underdiagnosed. Early ocular manifestations could be missed by ophthalmologists with limited experience. In this context, experts recommend raising awareness among pediatricians and ophthalmologists with respect to early signs of nephropathic cystinosis for timely diagnosis and adequate referral.

#### 4.1.3. CTNS Mutation Assessment

A wide variety of disease-causing *CTNS* mutations (at least 160 mutations) have been identified and associated with different disease presentations [16]. The *CTNS* mutation confirms the cystinosis diagnosis and can be used to diagnose unborn babies (antenatal screening in at-risk pregnancies) [5,17]. Recognizing the early symptoms of cystinosis in addition to corneal crystals helps ensure prompt diagnosis and treatment initiation for a better prognosis. Early signs and diagnosing factors include family history, glycosuria, and failure to thrive [17].

In the absence of WBC cystine level assays, 71% of experts check for *CTNS* mutations in addition to clinical and biochemical manifestations indicative of nephropathic cystinosis. The *CTNS* mutation test is readily available and fully covered by the different governmental institutions in the GCC with a turnaround time of 3 weeks.

### 4.2. Cystine-Depleting Therapy

Nephropathic cystinosis is a multi-system disease, warranting a multi-disciplinary management approach [18], with a medical board including nephrologists and ophthalmologists, as well as neurologists, endocrinologists, and gastroenterologists, consulted on a case-by-case basis.

#### 4.2.1. Immediate-Release (IR) Cysteamine

Cysteamine, also known as β-mercaptoethylamine, is an aminothiol regarded as the mainstay of therapy and treatment of choice for patients with cystinosis worldwide [19]. Cysteamine converts cystine to cysteine and cysteine-cysteamine, facilitating their exit from the lysosome in patients diagnosed with cystinosis [19]. The first immediate-release (IR)-cysteamine bitartrate formulation was approved by the FDA in 1994 for cystinosis treatment. While it has been shown to reduce WBC cystine levels by up to 95% and to reduce cystine levels in organ tissues, delaying the disease progression, IR-cysteamine is applied with a strict schedule, including nocturnal administration, every 6 h (Q6H), and is associated with side effects, including gastrointestinal adverse events and halitosis [20]. These challenges lead to suboptimal adherence and poor clinical outcomes in patients with cystinosis [21,22]. Since the cornea is avascular, cystine crystals are not resolved by oral cysteamine, thus topical cysteamine eye drops must also be applied. Photophobia and eye discomfort usually improve or resolve within weeks after starting eye drops (initially administered 4 times per day) [23]. Oral and eye drop cysteamine should be applied early and continuously to ensure better disease management and alleviate disease complications.

While kidney failure is cured by kidney transplant, it has no effect on the multi-systemic complications of cystinosis [5]. Hence, CDT has to be taken for life.

Cysteamine cannot reverse renal Fanconi syndrome, but has been shown to improve growth and to preserve kidney and extra-renal organ function in patients with cystinosis when applied before 5 years of age and to prolong life irrespective of the age of treatment initiation [1,5,20,21].

The frequency of pill intake (Q6H) and halitosis are considered the major obstacles to treatment adherence. According to the literature, around 85% of patients below 11 years of age and less than 50% of patients above 11 years of age adhered to eye drops and oral cysteamine treatment [17]. All panelists consider the nighttime pill as the major factor compromising adherence to treatment in the GCC, followed by the frequency of pills as reported by 86% of the panelists and adverse effects (57% of experts). Experts monitor patient adherence to the treatment by biochemical assessment and clinical symptoms, including smelling halitosis.

Panelists underlined the importance of parent–child communication about the disease but also advised on educating them on potential side effects of the treatment. In addition, they recommend assisting patients, especially adolescents, to accept the side effects and manage their social life to boost their self-esteem. Experts were concerned about assessing the effectiveness of eye drops, given the variety of factors that should be considered in the slit-lamp examination scoring system, such as the thickness of the cornea. The short one-week validity of the opened vial [24], the difficulty in applying eye drops due to muscle weakness and restrictions in eye drop production are additional concerns that should be resolved.

#### 4.2.2. Delayed-Release (DR)-Cysteamine

Single-dose DR-cysteamine overcomes the challenges related to IR-cysteamine while maintaining therapeutic plasma cysteamine levels and WBC cystine control over 12 h, comparable to Q6H IR-cysteamine [17]. An open-label, randomized, controlled, crossover, pivotal phase III trial showed a non-inferiority of DR-cysteamine versus IR-cysteamine in terms of WBC cystine level control [25]. WBC cystine depletion was achieved and sustained in the long-term with a lower daily dose of DR-cysteamine versus IR-cysteamine (dose equal to approximately 70% of their usual dose of IR-cysteamine) [25,26]. DR-cysteamine was associated with an improved quality of life, alleviating the associated social burden [27]. Stable growth and kidney function were maintained over two years with DR-cysteamine treatment [27]. Table 1 below summarizes the different aspects of IR- versus DR-cysteamine. Noteworthy, both IR- and DR-cysteamine are available in the GCC, but access to DR-cysteamine is more restricted due to its elevated cost.

Real-world evidence showed that the switch to DR-cysteamine was driven by difficulties with nighttime administration, halitosis, difficulty reaching cystine targets, non-adherence with IR-cysteamine dosing schedule, and an unstable metabolic situation [28]. WBC cystine level and kidney function remained stable or improved following the switch from IR-cysteamine to DR-cysteamine in patients with preserved kidney function [27,28,29]. In addition, switching to DR-cysteamine showed an improved linear growth, especially in pediatric patients [27,29], and a reduced rate of hospitalizations and days spent in hospital [27,29], of adverse events including halitosis, body odor, and gastrointestinal adverse events, and of proton pump inhibitor use [26,30].

**Table 1:** IR-cysteamine versus DR-cysteamine.

KERRYPNX	IR-Cysteamine	DR-Cysteamine
**Composition**	Hard capsule containing cysteamine (50 or 150 mg) [31]	Capsule containing individually enteric-coated, pH-sensitive micro-granules of cysteamine (25 or 75 mg) [25,32,33]
**Mode of delivery**	Released into the stomach causing a 3-fold increase in gastric acid production (ulcerogenic)	Released into the small intestine: bypassing stomach [32,33], potentially reducing gastric acid production
**Dosage**	Dosing every 6 h, day and night [31]	Dosing every 12 h
	Up to 12 years old: 1.3 g/m^2^/day in four divided doses	Target maintenance dose: 1.3 g/m^2^/day in two divided doses [32,33,34]
	Over 12 years old and >50 kg: 2 g/day in four divided doses	Over 12 years old and >50 kg: 2 g/day in two divided doses
**Administration with food**	Digestive tolerance improved when taken with or just after food [30]	Taken without food (ideally fast 1 h before and 1 h after dosing) or with a small amount of food (preferably carbohydrate) [34]
	In children below 6 years, the content of the capsules should be sprinkled on food [31]	In children below 6 years, capsules should be opened, and the content sprinkled on recommended food or drink [34]
	Foods such as milk, potatoes, and other starch-based products seem to be appropriate for mixing with the powder [31]	~100 g of carbohydrates (applesauce/fruit jam) may be mixed with DR-cysteamine granules but frozen, dairy, high-fat, or high protein foods should be avoided [34]
	Acidic drinks (e.g., orange juice) should be avoided, as the powder tends not to mix well and may precipitate out [31]	DR-cysteamine may be mixed with 100–150 mL of acidic fruit juice (e.g., orange juice) or water [34]
**Adherence**	Poor adherence (adolescents and adults)	Better adherence [29] and quality of life [27]
**Adverse effects**	Gastrointestinal complaints, halitosis, and body odor	Less gastrointestinal complaints, halitosis, and body odor Less proton pump inhibitor therapy required [35]
		Less proton pump inhibitor therapy required [35]

IR-cysteamine: Immediate-release cysteamine; DR-cysteamine: Delayed-release cysteamine.

Panelists noticed a remarkable improvement with respect to patient adherence and quality of life with DR-cysteamine treatment in the GCC. This was tightly linked to the reduction in halitosis, a leading social burden. In this context, experts believe that a standardized validated questionnaire should be generated for accurate quality of life assessment of patients with nephropathic cystinosis. Some validated Arabic quality of life questionnaires, such as the EuroQoL-5 dimensions-5 levels (EQ-5D-5L) and the Patient Assessment of Care for Chronic Conditions (PACIC-5As), might be useful to report patient outcomes.

Some experts suggested starting with DR-cysteamine treatment rather than switching at a later stage, since DR-cysteamine has a comparable effectiveness with a considerable adherence to the treatment and quality of life improvement.

## 5. Local Practice in the GCC Region

### 5.1. Challenges in Nephropathic Cystinosis Management

Globally, nephropathic cystinosis, a chronic disease, inevitably presents challenges related to therapy adherence, and the situation in the GCC is no different. Unsatisfactory treatment adherence reduces therapy effectiveness [22]. Missing or delaying a dose of cysteamine causes cystine accumulation that might damage organs and warrant kidney transplantation [22]. Adherence to cysteamine is higher among young patients (<11 years) taken in charge by their parents compared to older patients who are responsible for their own care [17]. Adolescents’ rebellion and risk-taking behavior, self-image, and disease denial may pose considerable challenges for medication adherence in cystinosis. The transition from pediatric to adult care is a high-risk period, as it overlaps with kidney transplantation and the development of extra-renal manifestations of cystinosis [36]. In adult patients, non-adherence is predominantly unintentional, mostly due to forgetfulness and organizational issues (up to 58%) [21]. Adverse events are also major contributors to unsatisfactory treatment adherence and contribute to 50% adherence among patients aged 11 years and older [17]. In this context, assistance by nursing and psychology teams can enhance treatment adherence [37].

### 5.2. Expert Recommendations

**Figure 1:**
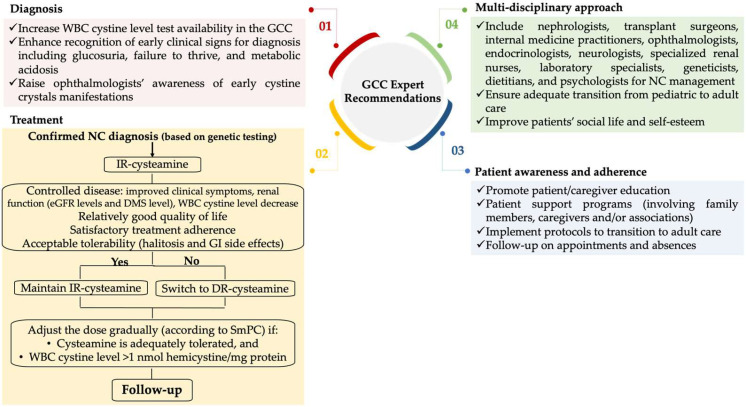
Practical guidance for optimizing the management of nephropathic cystinosis in the GCC. DR: delayed-release; DMS: dimethyl sulfoxide method; eGFR: estimated glomerular filtration rate; GCC: Gulf Cooperation Council; GI: gastrointestinal; IR: immediate-release; NC: nephropathic cystinosis; SmPC: Summary of Product Characteristic; WBC: white blood cells.

Experts identified gaps in the journey of patients with nephropathic cystinosis in the GCC and suggested an algorithm (Figure 1) that serves as guidance to achieve better disease control and attenuate disease manifestations, all while improving patients’ quality of life [17,33].

At the diagnosis level, experts hope to leverage accessibility to the WBC cystine test in the GCC and raise awareness among ophthalmologists for the timely diagnosis of cystine crystals, which could be indicative of nephropathic cystinosis.

Experts endorse maintaining IR-cysteamine when the disease is controlled in terms of clinical symptoms and kidney function with improved or maintained estimated glomerular filtration rate levels, a WBC cystine level within the recommended range, and acceptable quality of life and tolerability. Conversely, when the disease is progressing with unsatisfactory patient adherence, tolerability, and quality of life, experts recommend switching to DR-cysteamine. Since the patient plays a central role in the management of the disease, experts emphasize the importance of educating patients and caregivers in order to promote awareness about the disease, improve treatment adherence, and circumvent challenges. Finally, given the systemic nature of the disease with multi-organ involvement, experts urge a multi-disciplinary approach for optimal management of nephropathic cystinosis to treat associated complications and prevent kidney failure, to ensure an adequate transition between pediatric and adult care, and to improve social life, self-esteem, and overall well-being.

## 6. Conclusions

In the absence of international guidelines for nephropathic cystinosis management, this paper offers local guidance tailored to the GCC, aiming to fill the gaps and providing a unified and standardized approach for the nephropathic cystinosis patient journey. However, one limitation is that the management of extra-renal manifestations was not discussed in depth, as all experts were nephrologist and the discussion and questions predominantly focused on nephropathic cystinosis in the context of renal function. Future campaigns, professional networks, educational events, and publications will play a key role in raising awareness and promoting consistent clinical practices across the GCC.

This manuscript will serve as practical guidance to streamline the management of nephropathic cystinosis in the GCC region, the Arab world, and countries with similar patient profiles. Experts with experience in nephropathic cystinosis will continue fulfilling their responsibility to spread awareness among peers, as well as among patients and their caregivers.

Beyond the Middle East, international collaboration is essential to homogenize standards of care, foster shared learning, and support research efforts aimed at improving patient outcomes globally.

Regional and global guidelines for the diagnosis and management of nephropathic cystinosis will also benefit the medical and the patient communities.

## Data Availability

Participant responses to the survey questions are available on reasonable request.
